# Definition of Acute Respiratory Distress Syndrome on the Plateau of Xining, Qinghai: A Verification of the Berlin Definition Altitude-PaO_2_/FiO_2_-Corrected Criteria

**DOI:** 10.3389/fmed.2022.648835

**Published:** 2022-02-23

**Authors:** Xiaoqin Liu, Chun Pan, Lining Si, Shijun Tong, Yi Niu, Haibo Qiu, Guifen Gan

**Affiliations:** ^1^Department of Critical Care Medicine, Affiliated Hospital of Qinghai University, Xining, China; ^2^Jiangsu Provincial Key Laboratory of Critical Care Medicine, Department of Critical Care Medicine, Zhongda Hospital, School of Medicine, Southeast University, Nanjing, China

**Keywords:** acute respiratory distress syndrome, high altitude, Berlin Definition, ARDS, P/F

## Abstract

**Background:**

Acute respiratory distress syndrome (ARDS) is a common critical respiratory illness. Hypoxia at high altitude is a factor that influences the progression of ARDS. Currently, we lack clear diagnostic criteria for high-altitude ARDS. The purpose of this study was to determine the value of the application of the Berlin Definition altitude-PaO_2_/FiO_2_-corrected criteria for ARDS in Xining, Qinghai (2,261 m).

**Methods:**

We retrospectively analyzed the clinical data of patients with ARDS admitted to the Department of Critical Care Medicine of the Affiliated Hospital of Qinghai University from January 2018 to December 2018. The severity of ARDS was categorized according to the Berlin Definition, Berlin Definition altitude-PaO_2_/FiO_2_-corrected criteria, and the diagnostic criteria for acute lung injury (ALI)/ARDS at high altitudes in Western China (Zhang criteria). In addition, the differences between the three criteria were compared.

**Results:**

Among 1,221 patients, 512 were treated with mechanical ventilation. In addition, 253 met the Berlin Definition, including 49 (19.77%) with mild ARDS, 148 (58.50%) with moderate ARDS, and 56 (22.13%) with severe ARDS. A total of 229 patients met the altitude-PaO_2_/FiO_2_-corrected criteria, including 107 with mild ARDS (46.72%), 84 with moderate ARDS (36.68%), and 38 (16.59%) with severe ARDS. Intensive care unit (ICU) mortality increased with the severity of ARDS (mild, 17.76%; moderate, 21.43%; and severe, 47.37%). Twenty-eight-day mortality increased with worsening ARDS (mild 23.36% vs. moderate 44.05% vs. severe 63.16%) (*p* < 0.001). There were 204 patients who met the Zhang criteria, including 87 (42.65%) with acute lung injury and 117 (57.35%) with ARDS. The area under receiver operating characteristics (AUROCs) of the Berlin Definition, the altitude-P/F-corrected criteria, and the Zhang criteria were 0.6675 (95% CI 0.5866–0.7484), 0.6216 (95% CI 0.5317–0.7116), and 0.6050 (95% CI 0.5084–0.7016), respectively. There were no statistically significant differences between the three diagnostic criteria.

**Conclusion:**

For Xining, Qinghai, the altitude-PaO_2_/FiO_2_-corrected criteria for ARDS can distinguish the severity of ARDS, but these results need to be confirmed in a larger sample and in multicenter clinical studies.

**Clinical Trial Registration:**

ClinicalTrials.gov, identifier: NCT04199650.

## Introduction

Acute respiratory distress syndrome (ARDS) is a severe disease that has received considerable attention due to its high mortality rate (35–40%) (1). Proposed in 2012 (2), the Berlin Definition of ARDS is currently used to diagnose ARDS from the time of onset, the cause of pulmonary edema, and chest X-ray findings and stratify data according to PaO_2_/FiO_2_.

The Qinghai-Tibet Plateau, which has an average elevation exceeding 4,500 m, is the world's highest and China's largest plateau with an area of 2,500,000 km^2^. Nearly 6 million people live in Qinghai Province, and 3 million people live in high-altitude areas.

Due to the hypoxic conditions on the plateau, patients with ARDS have special pathophysiological changes and clinical manifestations (3). If the definition of ARDS is completely followed in the plateau area, it may lead to treatment errors in these patients. For example, the incorrect setting of mechanical ventilation parameters or the use of invasive mechanical ventilation in patients who would otherwise have been treated with non-invasive ventilation or a mask to improve oxygenation. In Zhang et al. (4) proposed the diagnostic criteria for acute lung injury (ALI)/ARDS at high altitudes in Western China, which has been widely used in these areas. However, the Zhang criteria are proposed according to the American-European Consensus Conference (AECC) standard, which has certain limitations. Since the Berlin Definition was proposed, the diagnostic criteria of ARDS for altitudes above 1,000 m should be updated.

For the plateau area, the Berlin Definition indicates that in areas with altitudes above 1,000 m, the PaO_2_/FiO_2_ should be calculated according to the correction formula [PaO_2_/FiO_2_ × (atmospheric pressure/760)] (2). However, there is no clear evidence to prove whether the “Berlin Definition altitude-PaO_2_/FiO_2_-corrected criteria” is suitable for patients with ARDS in high-altitude areas. The objective of this study was to update the definition using new data (epidemiological, physiological, and clinical trial data) to address the current limitations of the diagnostic criteria for ALI/ARDS at high altitudes in Xining in Western China and explore other defining variables.

## Materials and Methods

### Study Design

The study was a single-center, retrospective, observational study. The objective of the study was to verify the value of the application of the Berlin Definition altitude-PaO_2_/FiO_2_-corrected criteria for ARDS in Xining, Qinghai (altitude: 2,261 m). Because the data were de-identified, the hospital institutional review board waived the need for informed consent and approved the study. The trial was registered at clinicaltrials.gov (NCT04199650).

### Definition of ARDS

The Berlin Definition: All enrolled patients met the Berlin Definition of ARDS. PaO_2_/FiO_2_ is as follows: mild ARDS, 200 mmHg < PaO_2_/FiO_2_ ≤ 300 mmHg; moderate ARDS, 100 mmHg < PaO_2_/FiO_2_ ≤ 200 mmHg; and severe ARDS, PaO_2_/FiO_2_ ≤ 100 mmHg.

For the Berlin Definition altitude-PaO_2_/FiO_2_-corrected criteria (hereafter, altitude-PaO_2_/FiO_2_-corrected criteria; note that all patients met the Berlin Definition except for the PaO_2_/FiO_2_ criterion), the PaO_2_/FiO_2_ was calculated according to the plateau correction formula [PaO_2_/ ${\text{FiO}}_{2}^{*}$(barometric pressure)/760)]. PaO_2_/FiO_2_ was combined with the Xi'ning atmospheric pressure (581 mmHg), and the altitude-PaO_2_/FiO_2_-corrected criteria for ARDS severity are as follows: mild ARDS, 153 mmHg < PaO_2_/ FiO_2_ ≤ 230 mmHg; moderate ARDS, 76 mmHg < PaO_2_/ FiO_2_ ≤ 153 mmHg; and severe ARDS, PaO_2_/FiO_2_ ≤ 76 mmHg.

With the diagnostic criteria for ALI/ARDS at high altitudes in Western China [following Zhang criteria (4)], patients with ALI or ARDS were classified according to PaO_2_/FiO_2_, altitude (2,200 m), and the AECC diagnostic criteria. Patients with PaO_2_/FiO_2_ ≤ 150 mmHg were diagnosed with ARDS, whereas those with PaO_2_/FiO_2_ between 150 and 200 mmHg were diagnosed with ALI.

### Study Population

Patients who were undergoing mechanical ventilation and entered the Department of Intensive Medicine of the Affiliated Hospital of Qinghai University between January 2018 and December 2018 were screened. The patients had to meet the Berlin Definition altitude-PaO_2_/FiO_2_-corrected criteria, the Zhang criteria, or all of the above. We excluded patients younger than 18 years and those with an Intensive Care Unit (ICU) length of stay < 24 h.

### Data Collection

For every enrolled patient, clinical data were assessed to detect whether the patient fulfilled the Berlin Definition, the altitude-P/F-corrected criteria, the Zhang criteria, or all of the above. Demographic data, underlying diseases, risk factors for ARDS, and Acute Physiology and Chronic Health Evaluation (APACHE) II scores were recorded during the first 24 h of ICU admission. FiO_2_, blood gases, and illness severity were recorded on the first day. The first day was defined as the first day on which the patients met the ARDS or ALI criteria. Given the difficulty of comparing noninvasive ventilation settings with invasive modes, we excluded patients with noninvasive ventilation from the analysis of ventilator management. Interventions and treatments during the ICU stay and patient outcomes were recorded.

### Outcome Measures

The primary outcome measures were ICU mortality, 28-day mortality, and 28-day mechanical-ventilation-free days. The secondary outcome measures were the duration of mechanical ventilation, length of ICU stay, and length of hospital stay.

### Statistical Analysis

Normally distributed data are presented as the mean ± SD, and non-normally distributed data are presented as the median (interquartile range, IQR). *p*-values for categorical variables were calculated with the χ^2^-test, while *p*-values for continuous variables were estimated with the *t*-test, the Mann–Whitney test, ANOVA, or the Kruskal–Wallis test depending on the distribution and number of variables. The receiver operating curve (ROC) statistical analyses were performed by using GraphPad Prism for Windows version 8.1, and other statistical tests were performed with SPSS for Windows version 19.0. Statistical significance was assessed at the 2-sided *p* < 0.05 level.

## Results

Since the patients were from high-altitude areas, we analyzed the enrolled patients and their characteristics, blood gases, and mechanical ventilation data based on the altitude-P/F-corrected criteria.

### Characteristics of Enrolled Patients

**Figure 1:**
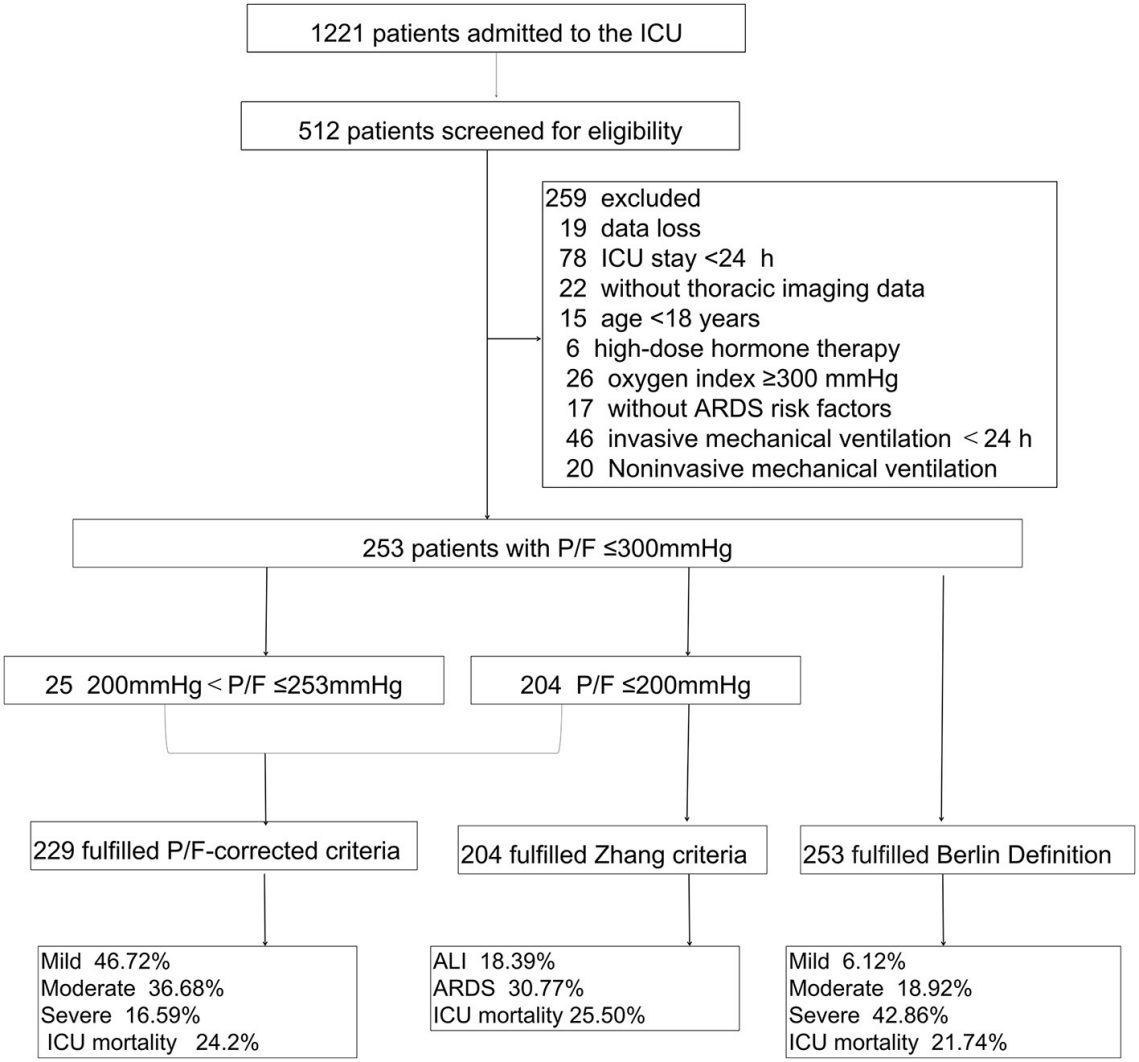
Flow diagram of patient screening and enrollment. P/F, PaO_2_/FiO_2_; P/F-corrected criteria, The Berlin Definition altitude-PaO_2_/FiO_2_-corrected criteria; Zhang criteria, The diagnostic criteria for ALI/ARDS at high altitudes in Western China; Berlin Definition, The Berlin Definition of acute respiratory distress syndrome; ALI, acute lung injury; ARDS, acute respiratory distress syndrome.

Among the 1,221 patients who were admitted to the ICU from January 1, 2018 to December 31, 2018, 512 had invasive mechanical ventilation after admission during the study period. Among them, 253 patients met the Berlin Definition, 229 met the altitude-P/F-corrected criteria, and 204 patients met the Zhang criteria (Figure 1).

### General Information

There were 229 patients who met the altitude-P/F-corrected criteria, of whom 107 (46.72%) had mild ARDS, 84 (36.68%) had moderate ARDS, and 38 (16.59%) had severe ARDS.

Patients with severe ARDS had fewer chronic hepatic dysfunctions than those with mild to moderate ARDS (*p* = 0.017), and APACHE II scores at presentation were slightly higher for patients with severe ARDS (*p* < 0.001). The most common risk factors were infection, pneumonia, aspiration, and trauma (Table 1).

**Table 1:** General information of 229 patients who met the altitude-P/F-corrected criteria.

	**Mild**	**Moderate**	**Severe**	***P* values**
No(%)	107(46.72%)	84(36.68%)	38(16.59%)	
Age (years, $\bar{\text{x}}$ ± SD)	50.45 ± 15.40	56.85 ± 16.83	59.05 ± 14.64	0.290
Male (n,%)	74(69.16%)	55(65.47%)	26(68.42%)	0.859
Source of ICU entry (n,%)				
Surgery	39(36.45%)	40(47.62%)	10(26.32%)	0.064
Internal medicine	10(9.35%)	10(11.90%)	12(31.58%)	0.003
Emergency	58(54.21%)	34(40.47%)	16(42.11%)	0.134
ARDS risk factors (n,%)				
Pneumonia	16(14.95%)	18(21.43%)	8(21.05%)	0.463
Infection	37(34.58%)	35(41.67%)	11(28.95%)	0.355
Aspiration	15(14.02%)	12(14.29%)	5(13.16%)	0.986
Trauma	22(20.56%)	15(17.86%)	4(10.53%)	0.383
Basic diseases (n,%)				
Hypertension	33(30.84%)	27(32.14%)	8(21.05%)	0.434
Diabetes mellitus	15(14.02%)	15(17.86%)	1(2.63%)	0.073
Chronic pulmonary disease	14(13.08%)	21(25.00%)	6(15.79%)	0.096
Chronic cardiac insufficiency	25(23.36%)	25(29.76%)	9(23.68%)	0.574
Chronic renal dysfunction	19(17.76%)	15(17.86%)	2(5.26%)	0.153
Chronic liver dysfunction	29(27.10%)	17(20.24%)	2(5.26%)	0.017
APACHE II score ($\bar{\text{x}}$ ± SD)	12.45 ± 5.11	13.24 ± 5.28	17.55 ± 5.52	<0.001
PaO_2_/FiO_2_ ($\bar{\text{x}}$ ± SD)	183.49 ± 20.04	118.15 ± 20.97	52.31 ± 12.39	<0.001
pH ($\bar{\text{x}}$ ± SD)	7.37 ± 0.31	7.29 ± 0.81	7.37 ± 0.11	0.572
PaCO_2_(mmHg, $\bar{\text{x}}$ ± SD)	36.52 ± 18.21	41.17 ± 18.26	42.10 ± 12.03	0.098
PaO_2_(mmHg,$\bar{\text{x}}$ ± SD)	82.25 ± 35.70	66.2 ± 22.92	43.86 ± 14.28	<0.001
FiO_2_(%,$\bar{\text{x}}$ ± SD)	63.92 ± 22.37	71.87 ± 21.41	89.47 ± 15.93	<0.001
PEEP (cmH_2_O,$\bar{\text{x}}$ ± SD)	5.11 ± 0.86	5.36 ± 1.59	5.26 ± 0.86	0.358
Lac (mmol/L, median IQR)	1.90(1.30–3.40)	2.40(1.40–4.85)	3.30(2.05–5.63)	0.030

*APACHE II, Acute Physiology and Chronic Health Evaluation II; PaO_2_, partial pressure of arterial oxygen; FiO_2_, fraction of inspired oxygen; PaO_2_, arterial oxygen partial pressure; PaCO_2_, arterial carbon dioxide partial pressure; FiO_2_, fraction of inspired oxygen; PEEP, positive end-expiratory pressure; Lac, lactic acid; IQR, Inter Quartile Range*.

### Blood Gases and Mechanical Ventilation Practices

Regarding pH indicators and arterial carbon dioxide partial pressure (PaCO_2_), the three groups had similar values. Lactate values (Lac) were significantly higher in patients with increased ARDS severity (Table 1).

The ventilator parameters were recorded during the first 24 h, during which the patients met the altitude-P/F-corrected criteria (Table 1). There was an absence of a statistical difference in applied positive end-expiratory pressure (PEEP) between the severity groups (*p* = 0.358). Patients with more severe ARDS had a higher ventilator setting for inspired oxygen concentration, and the difference was statistically significant (*p* < 0.001).

### Outcomes in Patients With ARDS

#### The Berlin Definition

A total of 253 patients met the Berlin Definition, of whom 49 (19.37%) had mild ARDS, 148 (58.50%) had moderate ARDS, and 56 (22.13%) had severe ARDS. The ICU mortality rates of patients with ARDS who had mild, moderate, and severe cases were 6.12, 18.92, and 42.86%, respectively (*p* < 0.001). With worsening ARDS, 28-day mortality increased (mild 6.12% vs. moderate 33.11% vs. severe 60.71%), and the median (IQR) number of ventilator-free days over a 28-day period decreased [mild ARDS, 26.17 (20.00–27.21) days; moderate ARDS, 18.73 (0.00–25.30) days; severe ARDS, 0.00 (0.00–18.26) days].

#### The Altitude-P/F-Corrected Criteria

Among the 229 patients who met the altitude-P/F-corrected criteria, 107 (46.72%) were diagnosed with mild ARDS, 84 (36.68%) were diagnosed with moderate ARDS, and 38 (16.59%) were diagnosed with severe ARDS. ICU mortality increased with the severity of ARDS (mild, 17.76%; moderate, 21.43%; and severe, 47.37%). Twenty-eight-day mortality increased with worsening ARDS (mild 23.36% vs. moderate 44.05% vs. severe 63.16%; *p* < 0.001). The median (IQR) number of ventilator-free days in a 28-day period declined with the severity of ARDS [mild, 21.42 (3.75–26.50) days; moderate, 16.53 (0.00–24.28) days; and severe, 0.0 (0.00–16.02) days (*p* < 0.001)].

The duration of mechanical ventilation in survivors slightly increased with the severity of ARDS [mild, 3.88 (1.04–9.13) days; moderate, 4.46 (1.40–9.45) days; and severe, 5.11 (2.73–11.85) days], but the difference was not statistically significant (*p* = 0.132). Differences between the length of ICU stay and the length of hospital stay across the Berlin Definition categories (mild, moderate, and severe) were statistically significant.

#### Diagnostic Criteria for ALI/ARDS at High Altitudes in Western China

There were 204 patients who met the Zhang criteria. Among the 204 enrolled patients, 87 (42.65%) had ALI and 117 (57.35%) had ARDS. The ICU mortality was 18.39% for those with ALI vs. 30.77% for those with ARDS (*p* = 0.045), and the 28-day mortality (51.28%) was higher for patients with ARDS than for patients with ALI (26.44%) (*p* < 0.001). The median (IQR) number of ventilator-free days over a 28-day period was higher in patients with ALI [19.50 (0.00–26.04)] than in patients with ARDS [4.21(0.00–23.31)] (*p* = 0.03). There were no significant differences in the length of ICU stay, the duration of invasive ventilation, and the length of hospital stay between patients with ALI and patients with ARDS (Table 2).

**Table 2:** Comparison of outcomes according to the three criteria.

	**Berlin Definition (253 cases)**				**Altitude-P/F-corrected criteria (229 cases)**				**Zhang criteria (204 cases)**		
	**Mild**	**Moderate**	**Severe**	***P* values**	**Mild**	**Moderate**	**Severe**	***P* values**	**ALI**	**ARDS**	***P* values**
No(%)	49(19.37)	148(58.50)	56(22.13)		107(46.72)	84(36.68)	38(16.59)		87(42.65)	117(57.35)	
ICU mortality, No(%)	3(6.12)	28(18.92)	24(42.86)	<0.001	19(17.76)	18(21.43)	18(47.37)	0.001	16(18.39)	36(30.77)	0.045
28-day mortality, No(%)	3(6.12)	49(33.11)	34(60.71)	<0.001	25(23.36)	37(44.05)	24(63.16)	<0.001	23(26.44)	60(51.28)	<0.001
No of ventilator-free days over a 28-day period, d, median (IQR)	26.17	18.73	0	<0.001	21.42	16.53	5.6	<0.01	19.5	4.21	0.03
	(20.00–27.21)	(0.00–25.30)	(0.00–18.26)		(3.75–26.50)	(0.00–24.28)	(0.00–16.02)		(0.00–26.04)	(0.00–23.31)	
ICU length of stay, d, median (IQR)	6	8.71	6.69	0.024	7.67	8.23	7.69	0.929	8.08	7.79	0.246
	(2.23–12.32)	(4.63–18.10)	(3.53–14.18)		(3.67–14.92)	(3.71–16.96)	(4.30–14.77)		(4.63–16.42)	(3.63–15.17)	
Duration of invasive ventilation, d, median (IQR)	1.79	4.75	4.23	0.003	3.88	4.46	5.11	0.132	5.08	4.67	0.945
	(0.73–4.98)	(1.50–10.62)	(2.16–9.92)		(1.04–9.13)	(1.40–9.45)	(2.73–11.85)		(1.46–9.83)	(2.15–10.63)	
Hospital length of stay, d, median (IQR)	24	22	16	0.001	23	18.5	18	0.036	23	18	0.059
	(19.00–38.00)	(13.25–35.75)	(6.00–25.75)		(14.00–37.00)	(9.25–30.00)	(8.00–26.00)		(14.00–38.00)	(9.00–29.50)	

*IQR, Inter Quartile Range*.

#### Area Under ROC Curve

**Figure 2:**
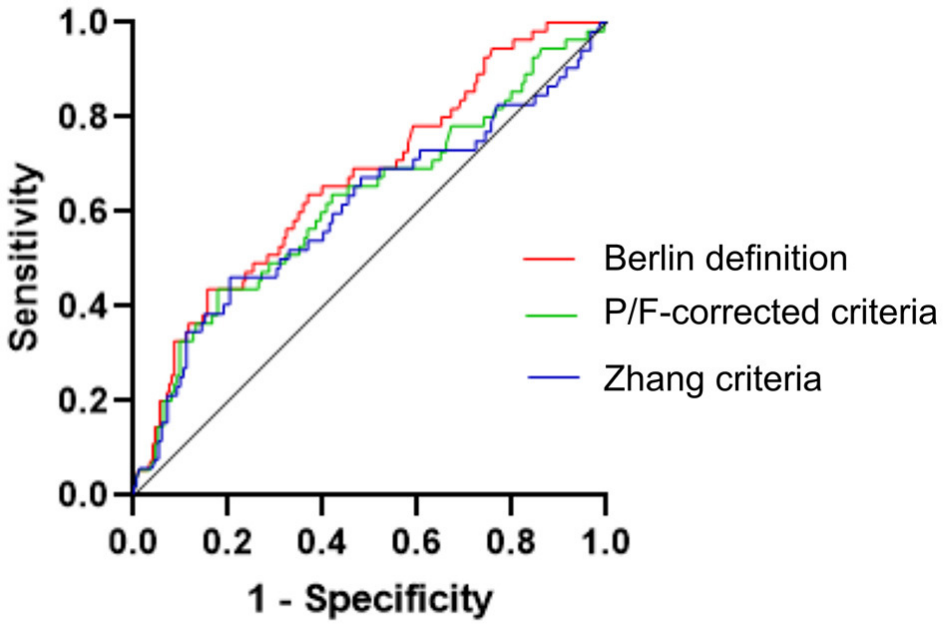
Comparison of the area under the receiver operating characteristic (ROC) curve of three different ARDS criteria for the ICU case fatality rate. Here were no statistically significant differences between the three diagnostic criteria. Berlin Definition, The Berlin Definition of acute respiratory distress syndrome; P/F-corrected criteria, The Berlin Definition altitude-PaO_2_/FiO_2_-corrected criteria; Zhang criteria, The diagnostic criteria for ALI/ARDS at high altitudes in Western China.

The area under the receiver operating characteristic (AUROCs) of the Berlin Definition, the altitude-P/F-corrected criteria, and Zhang criteria were 0.6675 (95% CI Power 0.5866–0.7484), 0.6216 (95% CI Power 0.5317–0.7116), and 0.6050 (95% CI power 0.5084–0.7016), respectively. There were no statistically significant differences between the three diagnostic criteria (Figure 2).

#### Characteristics of the Misclassifications of Patients With Altitude-P/F-Corrected Criteria and Berlin Definition

There were 24 patients with mild ARDS according to the Berlin Definition, but they did not meet the altitude-P/F-corrected criteria. All these patients were admitted to the surgery or emergency department and had almost no serious underlying diseases. In particular, only one patient had a history of chronic lung disease. The condition of these patients improved quickly after treatment, and all of them survived the ICU stay and were discharged from the ICU (Table 3).

**Table 3:** Characteristics of the misclassifications of patients with altitude-P/F-corrected criteria and Berlin Definition.

**Parameter**	**Patients with P/F 200–230**	**Patients with P/F 200–153**	**Patients with P/F 100–76**
No	24	82	18
APACHE II score ($\bar{\text{x}}$ ± SD)	8.67 ± 3.82	12.20 ± 5.31	4.22 ± 14.56
ICU mortality: No(%)	0	19.51%	33.33%
Duration of invasive ventilation, d, median (IQR)	1.75(0.70–8.06)	4.92(1.51–10.43)	3.15(1.05–4.29)
ICU length of stay, d, median (IQR)	6.75(2.98–14.61)	8.48(4.64–16.27	4.02(2.72–7.80)
Hospital length of stay, d, median (IQR)	26.5(20–40.25)	23(14.00–36.50)	14(5.00–20.75)
ARDS risk factors (n,%)			
Trauma	12(50)	19(23.17)	2(11.11)
Pneumonia	5(20.83)	12(14.63)	4(22.22)
Infection	12(50.00)	16(19.51)	3(16.67)
Aspiration	5(20.83)	11(13.41)	3(16.67)
Other	3(12.50)	24(29.27)	6(33.33)

*APACHE II, Acute Physiology and Chronic Health Evaluation II; IQR, Inter Quartile Range*.

There were 82 patients who met the Berlin Definition of moderate ARDS, but according to the plateau-P/F-corrected criteria, these patients were considered only to have mild ARDS. Sixteen of these patients died in the ICU (19.51%). Seventeen patients with severe ARDS, as defined by the Berlin Definition, met the altitude-P/F-corrected criteria of moderate ARDS, and the ICU fatality rate of these patients was 35.29%.

#### Characteristics of the Misclassifications of Patients With Altitude-P/F-Corrected Criteria and Zhang Criteria

There were 25 patients with mild ARDS according to the altitude-P/F-corrected criteria, but they did not meet the Zhang criteria. The oxygenation index of this part of patients is between 200 and 230 mmHg. Three of these patients died in the ICU (12%). Five patients with moderate ARDS, defined by the altitude-P/F-corrected criteria, met the Zhang criteria of ALI, and the ICU fatality rate of these patients was zero. The characteristics of these patients are shown in Table 4.

**Table 4:** Characteristics of the misclassifications of patients with altitude-P/F-corrected criteria and Zhang criteria.

**Parameter**	**Patients with P/F 200–230**	**Patients with P/F 200–153**
No	25	5
APACHE II score ($\bar{\text{x}}$ ± SD)	13.28 ± 4.14	15.6 ± 5.08
ICU mortality: No(%)	12%	0
Duration of invasive ventilation, d, median (IQR)	1.83(0.83–4.29)	6.79(1.08–8.08)
ICU length of stay, d, median (IQR)	4.42(1.96–9.42)	12.67(6.13–21.54)
Hospital length of stay, d, median (IQR)	24(16.00–35.00)	22(13.00–28.00)
ARDS risk factors (n,%)		
Trauma	3(12.00%)	2(40.00%)
Pneumonia	4(16.00%)	0
Infection	7(28.00%)	2(40.00%)
Aspiration	4(16.00%)	0
Other	7(28.00%)	1(20.00%)

*APACHE II, Acute Physiology and Chronic Health Evaluation II; IQR, Inter Quartile Range*.

#### The Distribution of Patients Across the Different Criteria

**Figure 3:**
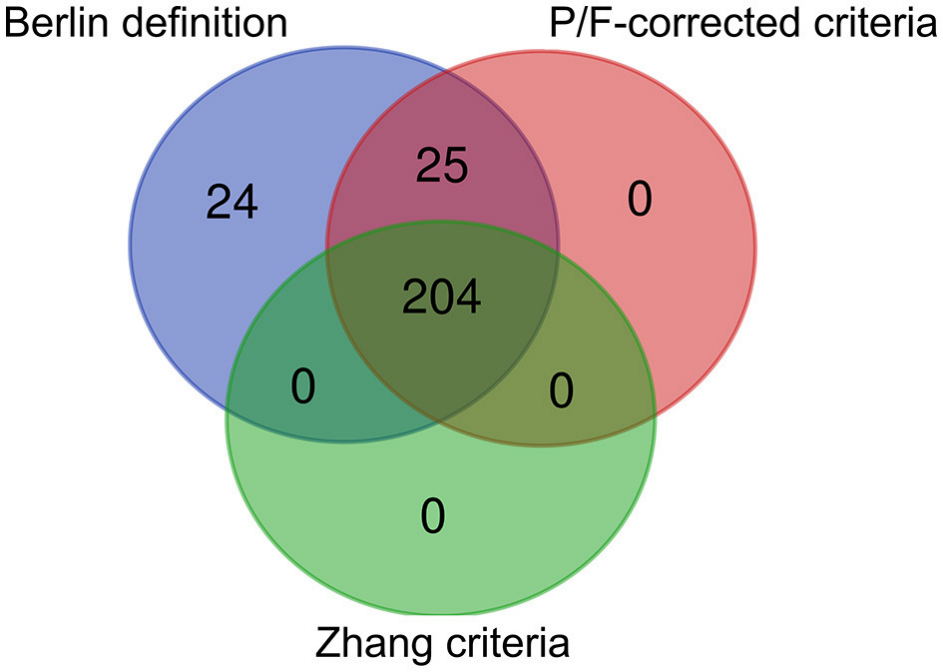
Venn diagram of patient distribution for different criteria. Berlin Definition, The Berlin Definition of acute respiratory distress syndrome; P/F-corrected criteria, The Berlin Definition altitude-PaO_2_/FiO_2_-corrected criteria; Zhang criteria, The diagnostic criteria for ALI/ARDS at high altitudes in Western China; The P/F was < 200 mmHg in 204 patients, 25 patients with a P/F between 200 and 253 mmHg, and 24 patients with 253 ≥ P/F < 300.

The P/F was < 200 mmHg in 204 patients who met both Zhang criteria and Berlin Definition and altitude-P/F-corrected criteria. There were 25 patients with a P/F between 200 and 253 who met the Berlin Definition and altitude-P/F-corrected criteria for mild ARDS. Twenty-four patients with only 253 ≥ P/F < 300 met the Berlin Definition and were considered to have mild ARDS (Figure 3).

## Discussion

The main manifestations of ARDS are persistent hypoxemia due to the pathophysiological changes of decreased lung volume (5), decreased compliance, and a decreased proportion of ventilatory blood flow (6). Although an increasing number of clinical studies in recent years have confirmed that some biomarkers are associated with ARDS (7), there is no evidence that they can be used in diagnosis (8, 9). The diagnosis of ARDS still focuses on the function of the lung, including the time of onset, oxygenation index, and imaging findings (10). P/F is the most important parameter to determine the severity of ARDS (11).

As mentioned earlier, high-altitude areas have characteristics of low oxygen pressure, strong radiation, cold climate, and so on (12). As altitude increases, the atmospheric pressure and the partial pressure of inhaled oxygen decrease (3). The low-oxygen environment in high-altitude areas has significant effects on the human body (13). Some studies have highlighted that the arterial blood partial pressure of healthy adults at high altitudes is significantly lower than that of healthy adults of the same age group in the plain area (14). Acute and chronic hypoxia at high altitudes can induce a variety of diseases and affect the quality of life and work capacity of people residing at high altitudes (15). People living at high altitudes for a long time have different tolerances for hypoxic environments, resulting in pathophysiological changes in patients with ARDS in high-altitude areas that are different from those in plain areas (16).

Studies have confirmed that ARDS associated with high altitude is characterized by hypoxemia that is difficult to treat and significantly increased pulmonary hypertension. In addition, the inflammatory response is more serious than what occurs in the plains. Hence, we need to update the criteria for ARDS in areas with altitudes above 1,000 m (17).

According to the AECC criteria, some patients with mild ARDS are diagnosed with ALI, and when clinicians lack awareness of this subgroup, they fail to provide appropriate support and treatment. In Zhang et al. (4) proposed the diagnostic criteria of ALI/ARDS in high-altitude areas of Western China on the basis of the AECC diagnostic criteria. Since then, the Zhang criteria have been widely used to diagnose ARDS in Western China. Since the Berlin Definition in 2012, the diagnosis criteria of ARDS in areas with altitudes above 1,000 m have not been updated. Therefore, we need to update the Zhang criteria (4). From this study, we know that when diagnosing patients with ARDS in Xining, Qinghai Province, according to the diagnostic criteria of ALI/ARDS in high-altitude areas of Western China, the case fatality rate of patients with ARDS is significantly lower than that of patients with ARDS in China and other parts of the world. This may be due to how the criteria exclude some patients with mild ARDS, that is, patients with P/F between 200 and 253 mmHg. Hence, no significant difference can be found between the groups.

According to the results of this study, the incidence rate of ARDS in the Affiliated Hospital of Qinghai University's intensive care unit was 16.70–20.72%, which is higher than that of the 2018 Large Observational Study to Understand the Global Impact of Severe Acute Respiratory Failure (LUNG SAFE) study (1, 18). This confirms that the incidence rate of ARDS increases in hypoxic environments at high altitudes. In the future, we will perform more clinical trials, which are necessary to confirm the findings of this study.

In this study, in Xining, Qinghai, the overall ICU mortality of patients diagnosed with ARDS according to the altitude-P/F-corrected criteria was 24.02%, and the ICU mortality of patients with mild, moderate, and severe ARDS was 17.76, 21.43, and 47.37%, respectively. The 28-day mortality was 37.56%, specifically, 23.36% for mild ARDS, 44.05% for moderate ARDS, and 63.16% for severe ARDS. The results are similar to the results of international and domestic large-scale epidemiological studies of patients with ARDS in recent years. In contrast, according to the Berlin Definition for the severity of ARDS, in Xining, Qinghai, the ICU mortality of patients with mild ARDS is only 6.12%, which is significantly lower than that reported in other epidemiological studies.

At high altitude, the oxygen content and partial pressure of oxygen in the air were lower than those in the plain area, and when the partial pressure of oxygen was < 300 mmHg in patients at high altitude, there were no other serious pulmonary pathological changes. Even if the patients had lung infiltration shadows and other characteristics consistent with an ARDS diagnosis, they could not be regarded as having ARDS.

The concept of ARDS has been put forward for 50 years, and progress in the care of patients with ARDS has been limited to refinement in supportive care such as lung protection ventilation, prone position, and even extracorporeal membrane oxygenation (ECMO). Delays or missed diagnosis of ARDS will cause clinicians to use wrong mechanical ventilation strategies, such as using the wrong PEEP, for patients. According to our research, patients with mild ARDS identified by the uncorrected Berlin Definition are very mild. Despite this, we think this is a false positive. These patients had a low APACHE II score and left the ICU after a short time with a good prognosis. In addition, they can quickly improve through routine oxygen therapy, therefore, these patients should not be diagnosed with ARDS.

According to the Berlin Definition, the length of ICU stay and the duration of invasive ventilation differed among the groups with mild, moderate, and severe ARDS. Despite this, no such difference was found according to the altitude-P/F-corrected criteria and Zhang criteria. After analyzing the results, we found that the between-group differences in the Berlin Definition were mainly due to the fact that the parameters of mild ARDS were very different from those of moderate to severe ARDS. This is also a false positive change caused by the inclusion of a large number of patients with the Berlin Definition.

This preliminary study confirmed that the altitude-P/F-corrected criteria can be used to classify the severity of ARDS in the Xining area of Qinghai Province. With the Zhang criteria, clinicians may fail to recognize the severity of ARDS in some patients, resulting in a delay in the diagnosis. On the other hand, the application of the Berlin Definition criteria in high-altitude areas may lead to a false-positive diagnosis of ARDS in a large number of patients, resulting in overtreatment.

This study has some limitations. 1) This study is a single-center retrospective study. Hence, the sample size is small and the sample source is limited, resulting in the sample not being very representative. The results must later be verified by multicenter clinical studies. 2) According to the results of this study, the altitude-P/F-corrected criteria for ARDS are applicable in Xining, Qinghai, but it is not clear whether these criteria are applicable in other areas at the same altitude or at other elevations, which must be verified by future multicenter clinical trials. 3) As there is no other strong basis for the diagnosis of ARDS at present, mortality was regarded as the main prognostic end point in this study, and in the process of disease progression, a variety of clinical intervention measures are often provided, which may affect the prognosis of patients to a certain extent. 4) In this study, we observed that there was no difference in the setting of PEEP at different severity levels. We collected the patient's first PEEP according to the Berlin definition of PEEP > 5 cmH_2_O, but since only the first PEEP was recorded. Due to this uncertainty, we cannot determine whether PEEP has an effect on the improvement in the P/F.

## Conclusion

For Xining, Qinghai Province, the Berlin Definition plateau criteria can distinguish the severity of ARDS in high-altitude areas, but these results need to be confirmed in a larger sample and in multicenter clinical studies.

## Data Availability Statement

The original contributions presented in the study are included in the article/supplementary material, further inquiries can be directed to the corresponding author.

## Ethics Statement

The study was a single-center, retrospective, and observational study. In order to verify the application value of Berlin Definition plateau criteria of ARDS in Xining, Qinghai (altitude:2261m). Because the data would be received in de-identified form (non-human subjects research), the Hospital Institutional Review Board waived the need for informed consent and approved the study.

## Author Contributions

CP and HQ designed the experiment. ST and XL collected the data. LS and YN analyzed the data. XL wrote the manuscript. CP and GG revised the manuscript. All authors contributed to the article and approved the submitted version.

## Funding

Support was provided solely from institutional and departmental sources. This work was supported by Qinghai Province Scientific and Technological Achievements Transformation Project (2019- SF-133), Environmental Medical Engineering Key Laboratory of the Ministry of Education Open Project (2020EME001), and Medical research Key project of Jiangsu Provincial Health Commission (ZD2021057).

## Conflict of Interest

The authors declare that the research was conducted in the absence of any commercial or financial relationships that could be construed as a potential conflict of interest.

## Publisher's Note

All claims expressed in this article are solely those of the authors and do not necessarily represent those of their affiliated organizations, or those of the publisher, the editors and the reviewers. Any product that may be evaluated in this article, or claim that may be made by its manufacturer, is not guaranteed or endorsed by the publisher.
